# Accuracy of steps measured by smartphones-based WeRun compared with ActiGraph-GT3X accelerometer in free-living conditions

**DOI:** 10.3389/fpubh.2022.1009022

**Published:** 2022-12-13

**Authors:** Qinqin Yao, Jing Wang, Yucong Sun, Li Zhang, Shuangyuan Sun, Minna Cheng, Qinping Yang, Siyuan Wang, Ling Huang, Tao Lin, Yingnan Jia

**Affiliations:** ^1^Key Lab of Public Health Safety of the Ministry of Education, School of Public Health, Fudan University, Shanghai, China; ^2^Shanghai Pudong New Area Center for Disease Control and Prevention, Shanghai, China; ^3^Winning Ringnex Technology (Shanghai) Co., Ltd., Shanghai, China; ^4^Shanghai Municipal Center for Disease Control and Prevention, Shanghai, China; ^5^Health Communication Institute, Fudan University, Shanghai, China

**Keywords:** ActiGraph-GT3X accelerometer steps, health-related outcomes, WeChat steps, free-living conditions, moderate-to-vigorous intensity physical activity

## Abstract

**Objectives:**

The purpose of this study was to evaluate the accuracy and reliability of steps tracked by smartphone-based WeChat app compared with Actigraph-GT3X accelerometer in free-living conditions.

**Design:**

A cross-sectional study and repeated measures.

**Methods:**

A total of 103 employees in the Pudong New Area of Shanghai, China, participated in this study. The participants wore an ActiGraph-GT3X accelerometer during the period of August to September 2019 (Time 1), December 2019 (Time 2) and September 2020 (Time 3). Each time, they wore the ActiGraph-GT3X accelerometer continuously for 7 days to assess their 7-day step counts. The smartphone-based WeRun step counts were collected in the corresponding period when subjects wore accelerometers. The subjects were invited to complete basic demographic characteristics questionnaires and to perform physical examination to obtain health-related results such as height, body weight, body fat percentage, waist circumference, hip circumference, and blood pressure.

**Results:**

Based on 103 participants' 21 days of data, we found that the Spearman correlation coefficient between them was 0.733 (*P* < 0.01). The average number of WeRun steps measured by smartphones was 8,975 (4,059) per day, which was higher than those measured by accelerometers (8,462 ± 3,486 per day, *P* < 0.01). Demographic characteristics and different conditions can affect the consistency of measurements. The consistency was higher in those who were male, older, master's degree and above educated, and traveled by walking. Steps measured by smartphone and accelerometer in working days and August showed stronger correlation than other working conditions and time. Mean absolute percent error (MAPE) for step counts ranged from 0.5 to 15.9%. The test-retest reliability coefficients of WeRun steps ranged from 0.392 to 0.646. A multiple regression analysis adjusted for age, gender, and MVPA/step counts measured during Time 1 showed that body composition (body weight, BMI, body fat percentage, waist circumference, and hip circumference) was correlated with moderate-to-vigorous intensity physical activity, but it was not correlated with WeRun step counts.

**Conclusions:**

The smartphone-based WeChat app can be used to assess physical activity step counts and is a reliable tool for measuring steps in free-living conditions. However, WeRun step counts' utilization is potentially limited in predicting body composition.

## Introduction

The physical activity guidelines issued by the Bull et al. (1) recommend that adults aged between 18 and 64 years engage in at least 150 min of moderate-to-vigorous aerobic physical activity per week (2, 3). However, more than 1.4 billion people failed to meet this standard, considered as physically inactive. Physical inactivity has become the fourth leading risk factor for human mortality after high blood pressure, tobacco use, and high blood sugar, resulting in more than 5 million deaths worldwide and an economic burden of at least USD 67.5 billion (2, 3).

The ability to measure physical activity scientifically, effectively, and accurately is an essential precondition for health epidemiology and intervention research (4). There are several ways to determine physical activity, including objective methods such as accelerometer, pedometer, and portable metabolic systems, as well as subjective recall questionnaires, such as the International Physical Activity Questionnaire (5) and physical activity logs (5).

As one of the most commonly used accurate measurement tools for measuring physical activity (6), the ActiGraph-GT3X accelerometer can measure steps, sedentary time, and time spent in moderate-to-vigorous intensity physical activity (7). It has been used as a gold standard to evaluate the validity of other measurements of physical activity levels (8). However, accelerometers are difficult to be widely used by the general public to measure steps because they are expensive and require technical expertise, specialized hardware, and cannot be worn for a long time, and the measurement results cannot provide immediate feedback (9).

Walking is one of the most popular types of moderate intensity physical activity. It has substantial importance to decrease chronic disease (such as obesity and CVD) and reduce medical expenditures (10). Step counts taken in daily life are a basic parameter of physical activity evaluation (11). Moreover, steps measured by devices are objective and intuitive, and using this measurement indicator to evaluate the standard of physical activity is quite suitable for the public to understand. Steps are widely accepted by researchers, practitioners, and the general public for assessing, tracking, and communicating the amount of physical activity (12–14).

WeChat, which was researched and developed by Tencent, is the most popular multi-purpose social networking platform in China, with about 1.1 billion monthly active users in 2018 (15). WeRun is an official account with step-counting function embedded in the WeChat app. After following WeRun, customers can obtain the step counts they take at any time measured by built-in accelerometer of their smartphones and share the step counts over the cloud through a secure server (16). WeRun in WeChat app is promising and cost-effective in step measuring, because it allows users to access their data anytime, anywhere (17). Christoph et al. (18) pointed out that short and intermittent bouts of activity may cause inaccuracies in the smartphone-counted steps, thus limiting the validity of smartphones in unconstrained conditions. The previous studies provided useful reference to the validity of devices used to measure physical activity, but they have several limitations: first, the reliability of some research was assessed under laboratory conditions but not free-living environments (19–21). In addition, these studies were based on cross-sectional data collection and the credibility of these findings needs further longitudinal research (22, 23). Physical activity in a short period of time cannot fully represent the long-term physical activity levels. It is well known that physical activity is not constant (24). Present studies have found that some factors can influence physical activity levels, including gender, age, seasons, and travel modes. For instance, active modes of transportation, such as walking, cycling, and public transportation, are associated with more steps and energy expenditure than personal motor vehicle travel (25, 26). These factors may affect the accuracy of the WeChat-counted steps.

Therefore, our objectives for this study were to (1) verify the consistency of the smartphone-based WeRun steps and the Actigraph-GT3X accelerometer-counted steps of the same subjects under different characteristics and conditions (travel modes, seasons, and weekday/weekend) through measurements of seven consecutive days at multiple time points (2) examine the test-retest reliability of the WeRun steps and (3) compare the predictive value of WeRun steps and moderate-to-vigorous intensity physical activity (MVPA) measured by the ActiGraph-GT3X accelerometer for health-related outcomes.

## Methods

### Sample size

The total sample of the study was determined by using a single population formula by assuming a 5% level of significance, 0.3 margin error and taking 35% proportion of physical inactivity. Considering a 20% non-response rate, the final sample size was 100.


$$\begin{array}{l} \text{N}=\frac{{P\;\left(1-\;P\right)\;Z^{2}}_{1-\;\alpha /2}}{d^{2}} \end{array}$$

### Participants

In this study, 103 participants were included from eight workplaces in the Pudong New Area of Shanghai, China. The inclusion criteria were as follows: (a) healthy adults without physical disabilities or diseases that impede movement; (b) own and regularly use a smartphone that they are willing to use to register a WeChat account and follow the WeRun official account; and (c) voluntary participation. The exclusion criteria were the following: (a) employees with heart or mental illness who are not suitable for exercise (based on self-report); (b) pregnant women; and (c) employees who intend to resign from their current workplace within 1 year. Eligible participants were provided with detailed information about the purpose and procedures of the study, and signed their informed consent. All study procedures were approved by the Shanghai Municipal Center for Disease Control and Prevention Ethical Review Committee (ChiCTR1900023813).

### Measurements

A cross-sectional study and repeated measures was conducted. ActiGraph-GT3X accelerometers were worn during the period of August to September 2019 (Time 1), December 2019 (Time 2), and September 2020 (Time 3), each time for 7 days (five workdays and two weekends). Smartphone-based WeChat application-counted steps of the participants in the corresponding time period were collected. Daily step counts were measured by WeRun, which is a social fitness plugin built in WeChat (informed consent of subjects). The ActiGraph-GT3X accelerometer was used to measure the physical activity of the subjects for seven consecutive days, including step counts and levels of moderate-to-vigorous physical activity. The original data of the accelerometers was collected at frequency of 30 Hz. Before the test, the accelerometer was initialized; the correct way of wearing the accelerometer and matters needing attention were introduced; and informed consent was signed.

Specific requirements for wearing it are as follows: (a) the accelerometers are fixed at the waist and positioned on another axillary line at the iliac crest level of the right or left hip (equipped with a flexible and adjustable elastic belt); (b) time of wearing: the accelerometer should be worn for seven consecutive days except during sleeping, bathing, or swimming. The accelerometers recorded activity during the day, and were removed at night. If the number of days is <3 days a week or the time of wearing is <8 h a day, the data is invalid. The ActiGraph-GT3X accelerometer data were extracted at an interval of 60 s.

The participants followed the official account of WeRun and completed registration on the WeChat online platform. They checked-in on the online official account of WeChat. The WeRun platform can obtain the daily data of the participants' WeRun step counts. The days with <1,000 steps were considered as invalid wearing days, and steps were truncated at 30,000 steps/day. In addition, each time the accelerometer was issued, participants were asked to fill out a questionnaire and undergo a physical examination. The data were collected by trained research assistants. The content of the questionnaire mainly included demographic characteristics (birth year, gender, age, marital status, education level, years of work, and travel modes used in the last week). Physical examination comprised height, weight, body fat percentage, waist circumference, hip circumference, systolic blood pressure, and diastolic blood pressure. This study assumed that the height of the participants did not change during the study. The participants' height was only measured once during inclusion in the study. Height was measured to the nearest 0.1 cm, and body weight was measured to the nearest 0.1 kg. Participants were required to be barefoot when measured for height. Height and weight were measured using the TCS-150 electric scale and Omron HBF-214 Body Composition Monitor Scale, respectively, and body fat percentage was measured using the reliable and valid Omron HBF-214 Body Composition Monitor Scale (Omron based on bioelectrical impedance technology is frequently used to measure body composition) (27). Blood pressure was measured by an electronic sphygmomanometer (Citizen, Model PW332) after the participants remaining relaxed for 2 min. The body mass index (BMI) was calculated by dividing an individual's weight in kilograms by the square of their height in meters (kg/m^2^).

### Data analysis

ActiLife 6.1.4 is a specialized software for ActiGraph-GT3X accelerometer data processing. The test data of step counts were downloaded to a computer at 60 s intervals and moderate intensity physical activity and vigorous intensity physical activity were downloaded at 10 s intervals (6, 28) through ActiLife 6.1.4, then added them up to MVPA [Based on cut points (29)]. WeRun imports step count data to Microsoft Excel^®^ 2019 from a smartphone's built in accelerometer, and during the study participants share their daily steps *via* a cloud-based secure server. The data were subsequently analyzed in IBM SPSS version 25.0 and SAS version 9.4. The descriptive statistics of the basic demographic characteristics were expressed in terms of proportion (%) and Mean (SD). Bland-Altman plot was used to examine the agreement on step counts measured by smartphone-based WeRun and accelerometer (steps/day). The Shapiro–Wilk test was used to test the normality of continuous variables, and the Spearman correlation coefficient was calculated to determine the relationship between the number of WeRun steps/day and the number of accelerometer steps/day. Mean absolute percentage error (MAPE, [estimated values–measured values]/measured values × 100%) and paired samples *t*-test were calculated to quantify the differences between the smartphone-based WeRun and accelerometer (the criterion measures) at the individual level. Pearson correlation analysis was conducted to calculate the intra class correlation that can be defined as the degree of consistency among three periods. Multivariate regression analysis was used to determine the correlation between health-related outcomes (body weight, BMI, body fat percentage, waist circumference, hip circumference, and blood pressure) and moderate-to-vigorous intensity physical activity and WeRun step counts (adjusted for age, sex, and MVPA or step counts measured by Time 1). MVPA, as well as Step counts measured by WeChat app and accelerometers, were acquired during each period and the three measures were used to calculate the coefficient of association, paired samples *t*-test and regression analysis. *P* < 0.05 was considered statistically significant.

## Results

### Demographic characteristics of the participants

The demographic characteristics of the participants are shown in Table 1. A total of 103 employees participated the study with mean age of 39.4 ± 10.4 years, nearly two thirds of the participants were female, more than 75% had a college junior degree or higher, and more than four in five were married. At baseline, less than half of the participants were classified as normal weight, 41.7% overweight and 14.6% obese based on BMI risk classification specific to Asian populations. Based on body fat percentage and blood pressure, more than 50% were classified at higher risk.

**Table 1:** Participants characteristics at baseline.

**Variables**	***n*** **(%)/mean (SD)**
**Gender**	
Male	34 (33.1)
Female	69 (66.9)
**Age (year)**	
≤ 35	40 (38.8)
35–45	33 (32.1)
>45	30 (29.1)
**Marital status**	
Single/divorced/widowed	19 (18.5)
Married	84 (81.5)
**Working year**	
≤ 5	44 (42.7)
5–15	34 (33.1)
>15	25 (24.2)
**Education**	
High school graduate or below	16 (25.2)
Junior college and Undergraduate	61 (59.3)
Master degree or above	16 (15.5)
Body weight	62.7 (11.0)
**Body Mass Index (Kg/m** ^**2**^ **)**	
Low risk (< 23)	42 (40.8)
Increased risk (23–27.4)	43 (41.7)
High risk (≥27.5)	15 (14.6)
**Body fat percentage**	
Lower risk (≤ 20% for men and ≤ 30% for women)	35 (34.0)
Higher risk (> 20% for men and > 30% for women)	67 (65.0)
Waist circumference (cm)	80.7 (9.3)
Hip circumference (cm)	95.2 (6.6)
**Blood pressure**	
Lower risk (SBP < 120 mmHg and DBP < 80 mmHg)	50 (48.5)
Higher risk (SBP ≥120 mmHg or DBP 80 ≥mmHg)	52 (50.5)
Moderate-to-vigorous intensity physical activity (MVPA) (min/day)	37 (23)

### Influencing factors of the correlation coefficient

**Figure 1:**
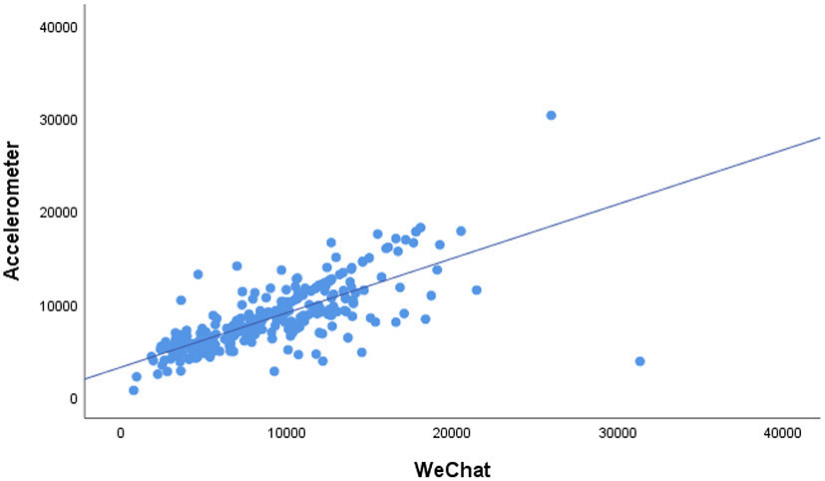
Scatter plot of the accelerometer and WeRun steps/day.

**Figure 2:**
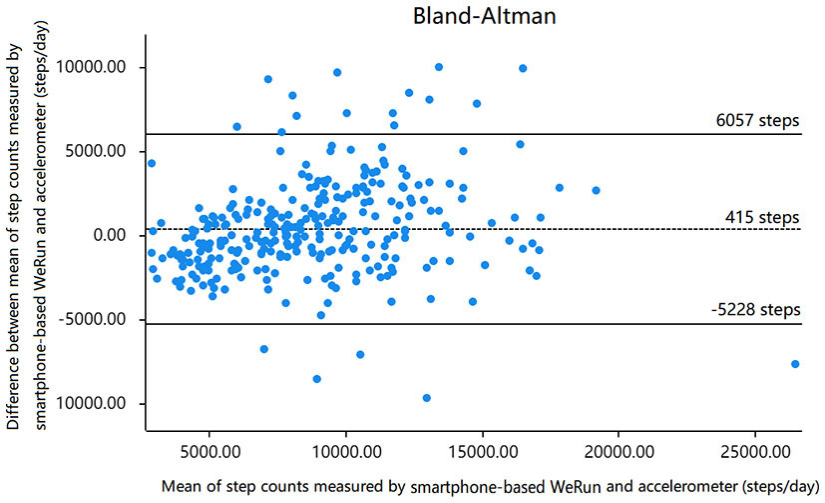
Agreement on step counts measured by smartphone-based WeRun and accelerometer (steps/day).

Figure 1 shows the scatter plot of the average daily steps measured by the ActiGraph-GT3X accelerometer and the WeChat app. It can be seen from the scatter plot that there was a significant positive correlation between the ActiGraph-GT3X accelerometer and WeChat app. Similarly, correlation between WeRun and accelerometer was found in Bland-Altman (Figure 2). As shown in Table 2, the average step counts per day measured by the WeChat app and ActiGraph-GT3X accelerometer were 8,974 (4,203) and 8,462 (3,486), respectively. The overall correlation coefficient between the accelerometer and WeRun was 0.733 (*P* < 0.01).

**Table 2:** Influencing factors of the correlation between WeRun step counts and ActiGraph-GT3X accelerometer step counts.

**Variables**		**WeRun steps/** **mean (SD)**	**Accelerometer steps/** **mean (SD)**	***r***	**MAPE (%)**
	General	8,975 (4,059)	8,462 (3,486)	0.733^**^	6.1^**^
Gender	Male	10,343 (3,250)	9,290 (2,703)	0.827^**^	11.3^**^
	Female	8,472 (3,619)	8,071 (2,763)	0.787^**^	5.0
Age (year)	≦35	8,668 (3,431)	8,219 (2,890)	0.735^**^	5.5
	35–45	10,077 (5325)	9,108 (4,060)	0.782^**^	10.6^*^
	>45	8359 (4,302)	8073 (3354)	0.799^**^	3.5
Education	High school and below	11,897 (4,174)	10,727 (3,261)	0.835^**^	10.9^**^
	Junior college and Undergraduate	7,790 (2,838)	7,511 (2,221)	0.770^**^	3.7
	Master degree or above	9,368 (2,478)	8,404 (1,701)	0.859^**^	11.5^*^
Working year	≦5	8,136 (2,835)	8,098 (2,362)	0.789^**^	0.5
	5–15	10,416 (4,076)	9,310 (3,661)	0.937^**^	11.9^**^
	>15	8,891 (3,681)	7,948 (2,794)	0.780^**^	11.7^**^
Travel mode	Walking	10,247 (1,128)	9,237 (859)	0.932^**^	10.9^**^
	Bicycle	9,489 (1,298)	9,714 (837)	0.857^**^	2.3
	Public transportation	8,364 (463)	7,959 (316)	0.927^**^	5.1
	Personal motor vehicle	8,400 (761)	7,713 (475)	0.548^*^	8.9
Working status	Weekend	7,792 (3,647)	7,434 (2,820)	0.752^**^	4.8
	Weekday	9,636 (3,880)	8,844 (3,104)	0.835^**^	9.0^**^
Month	August	9,373 (5,831)	9,620 (5,887)	0.998^**^	2.6
	September	8,182 (5,835)	7,759 (4,111)	0.762^**^	5.5^**^
	December	6,455 (4,494)	7,674 (3,947)	0.778^**^	15.9^**^

* *P* < 0.05,

** *P* < 0.01 (two-tailed). In both cases (* and **), the correlation and paired samples t-test was statistically significant.

The correlation coefficient is different for different demographic characteristics and different conditions. There was a stronger consistency in males (*r* = 0.827, *P* < 0.001) and people who were over 45 years old (*r* = 0.799, *P* < 0.001). The correlation was the weakest (0.770) among college and bachelor's degree graduates compared with those with high school or below (0.835) certifications and master's degree or above (0.859). The correlation between working years from 5 to 15 years was the strongest (0.937). Participants who travel by personal motor vehicle showed the weakest correlation, at just 0.548. The correlation coefficient on weekdays (0.835) was higher than that on weekends (0.752). The correlation between the accelerometer steps and the WeRun steps was also affected by time, with August at 0.998, September at 0.762 and December at 0.778. Mean absolute percent error (MAPE) for step counts ranged from 0.5% to 15.9%. The magnitude of MAPE was highest for December (Table 2).

### Reliability estimate

For test-retest reliability, the intraclass correlation coefficients ranged from 0.392 to 0.646. The details are shown in Table 3. WeRun steps measured in Time 1 and Time 2 (*r* = 0.646) have higher retest reliability than Time 1 and Time 3 (*r* = 0.478), Time 2 and Time 3 (*r* = 0.392).

**Table 3:** Reliability of the WeRun steps.

**Evaluation index**		**WeRun steps** **(Time 1)**	**WeRun steps** **(Time 2)**	**WeRun steps** **(Time 3)**
WeRun steps (Time 1)	ICC	1		
WeRun steps (Time 2)	ICC	0.646^**^	1	
WeRun steps (Time 3)	ICC	0.478^**^	0.392^**^	1

** *P* < 0.01 (two-tailed).

ICC, Intraclass correlation coefficient.

### Regression analyses of moderate-to-vigorous intensity physical activity and smartphone-based step counts with health-related outcome

MVPA was measured by the ActiGraph-GT3X accelerometer. Table 4 presents the results of multiple regression analysis that we used to examine the predictive relationships among WeRun steps and MVPA with health-related outcomes (weight, BMI, body fat proportion, etc.). Health-related outcomes were considered the dependent variables and WeRun steps/MVPA were considered the independent variable. We controlled for the effects of gender, age, and WeRun steps/MVPA at baseline. Body composition showed a significant negative association with high levels of MVPA. The results suggest that each additional minute of moderate-to-vigorous intensity physical activity can reduce body weight by 0.14 kg, BMI by 0.053, body fat percentage by 0.053, waist circumference by 0.119 cm, and hip circumference by 0.090 cm. However, no significant relationships between WeRun step counts and health-related outcomes (body weight, BMI, body fat percentage, waist circumference, hip circumference, waist–hip ratio, and blood pressure) were observed (*P* > 0.05).

**Table 4:** Multiple regression analysis of health-related outcomes and WeRun step counts/MVPA.

**Variables**	**WeRun steps**	**MVPA**
	**B (95% CI)**	**B (95% CI)**
Weight (Kg)	−2.563 (−7.196–2.071)	−0.14 (−0.246 −0.035)^*^
BMI	−1.251 (−2.924–0.422)	−0.053 (−0.091−0.014)^*^
Body fat percentage	−1.206 (−3.174–0.762)	−0.053 (−0.099 −0.007)^*^
Waistline (cm)	−1.598 (−4.820–4.500)	−0.119 (−0.211 −0.026)^*^
Hipline (cm)	−2.886 (−6.025–0.54)	−0.090 (−0.162 −0.017)^*^
Waist-hip ratio	0.009 (−0.16–0.034)	< 0.001 (−0.001– < 0.001)
SBP (mmHg)	−3.121 (−10.313–4.070)	0.003 (−0.169–0.176)
DBP (mmHg)	−0.158 (−5.388–5.072)	−0.004 (−0.125–0.118)

* *P* < 0.05 (the regression coefficient was statistically significant).

## Discussion

The results of this study indicated that the step counts estimated by the smartphone-based WeChat application were generally consistent with the step counts obtained by the ActiGraph-GT3X accelerometer, with a correlation coefficient of 0.733 (*P* < 0.01). Steps measured by the WeRun app were higher by 513 steps per day than those measured by the ActiGraph-GT3X accelerometer (*P* < 0.01). To our knowledge, this is the first longitudinal study on the accuracy of the smartphone-based WeChat app step counts for collecting different demographic characteristics under different conditions. Our study results are consistent with those of Victor et al. (30) and Janaine et al. (22). The results show that WeRun steps measured by smartphone are highly correlated with accelerometer-measured steps. However, WeRun overestimated steps generally, and whether WeRun steps can be used to evaluate the steps precisely is affected by some factors, such as gender, age, education, working years, working status and seasons, etc. (31). However, these findings are contrary to those of some studies, such as Höchsmann et al. (18) and Piccinini et al. (13). These latter studies argued that smartphone-based apps were unreliable for measuring step counts. However, it should be noted that the wearing time and measuring environment could cause differences among studies. Those studies' data were collected in specific conditions (running machine, playground, corridor, etc.) and not in a free-living environment. Moreover, participants' wearing time was no more than 8 h per day, and some individuals just wore the accelerometer for several minutes every time. These were possible reasons for the apparent discrepancies between our findings and those of the aforementioned studies (32). Our study found that the step count correlation between the smartphone-based WeChat application and the ActiGraph-GT3X accelerometers was not constant under different users' characteristics and conditions. We found that gender, age, modes of travel, working status, and other factors can affect the consistency and accuracy of the WeChat app and the Actigraph-GT3X accelerometer.

With the popularity of smartphone-based WeRun step counts in research and practice, accuracy and precision are critical, especially under diverse conditions. According to our findings, the correlation coefficient is higher in males than females (0.827 vs. 0.787, respectively). The number of WeRun steps of males was more than that of females and the consistency was stronger in males, which may be due to wearing time and wearing position. For example, females took fewer steps than men regardless of age, a finding that may be partly due to differences in mobile phone carrying habits and locations. Females' apparels, especially dresses, rarely have pockets for smartphones. Most females have the habit of carrying their mobile phones in their bags when they go out. The positioning of devices for monitoring steps will affect the accuracy of the devices (33, 34). The correlation was stronger when mobile phones were placed closer to the body during longer daytime activities (32, 33). With the increase of age, the correlation between the WeRun steps and the accelerometer steps gradually increased. It may be related to changes in lifestyles and intensity of their physical activity with age (35).

In addition to the characteristics of the subjects, different conditions may also affect consistency. Compared with working days, the number of steps were lower and the correlation coefficient was weaker than weekends. It may be that the time of carrying smartphones and wearing the ActiGraph-GT3X accelerometer was different between weekdays and weekends, thus causing the correlation between the ActiGraph-GT3X accelerometer and the WeChat app on weekends to be lower than that on weekdays (36). For the different travel modes, the consistency of walking was best, and personal motor vehicle had particularly weak correlation due to the location of the mobile phones (37).This is due to step-counting principle of built-in accelerometer of smartphone, which is affected by the location of the phone. Therefore, it was not accurate and precise to evaluate WeRun steps when traveling by a personal motor vehicle.

The test-retest reliability coefficients of the WeRun steps ranged from 0.392 to 0.646. WeRun steps measured in Time 1 and Time 2 (*r* = 0.646) have higher retest reliability than Time 1 and Time 3 (*r* = 0.478), Time 2 and Time 3 (*r* = 0.392). This may be that several-month intervals for the test-retest reliability were selected in this study. Besides, previous studies documented that walking behavior is affected by the COVID-19 and weather (16, 38). This study was conducted in summer and winter, and the weather effects (e.g., rain, wind) during the data-collected would limit people's travel to a certain extent (36). The third point in time for data collection occurred after the COVID-19, and walking behavior can be highly variable (16). Therefore, objective factors such as time and epidemic situation may hinder the reliability of WeRun steps.

Steps measured by ActiGraph-GT3X accelerometer and smartphone are consistent, but there are differences in the predictive value of body composition. Multivariate regression analysis controlling age, gender, and MVPA/WeRun steps at baseline showed that moderate-to-vigorous intensity physical activity can affect body composition, such as weight, BMI, body fat percentage, waist circumference, hip circumference, and waist–hip ratio (*P* < 0.05), but the WeRun step counts could not. This may because the ActiGraph-GT3X accelerometer has the function of distinguishing the intensity of physical activity, whereas WeRun step counts do not. It might be due to a measurement gap during exercising or other vigorous activities performed without the smartphone (39). Besides, smartphones with a built-in accelerometer can only track steps according to a user's movement and cannot monitor the user's type and quality of movements performed, such as jogging and walking, even though jogging consumes more energy than walking (40). As a result, there is no statistical significance in the correlation between body composition and the WeRun step counts. Although the step measurements are similar between the WeChat app and the accelerometer, WeRun steps cannot replace the role of the accelerometer in predicting body composition. The accelerometer not only tracks the number of steps but also monitors different intensities of physical activity and their duration, thus providing a more direct and clear assessment of energy expenditure. The results of a meta-analysis by Hamer et al. suggested that moderate-to-vigorous intensity physical activity is more effective in improving body composition than low-intensity physical activity (41). However, WeRun-measured step counts have a good prospect in long-term monitoring and supporting a beneficial change in the trend of the population's physical activity.

As economic and technological advances increase the focus on health, devices that track physical activity, such as pedometers, are steadily improving in accuracy and precision. However, owing to the impacts of price, battery life, comfort, applicability, water resistance, and other factors, the use of step-recording equipment by the public is limited (42). WeChat, which is one of the most popular social apps in China, has a feature to track the number of steps. However, the smartphone-based WeChat app cannot assess the duration and intensity of physical activities. In fact, how to improve the accuracy of step counts is still a very important challenge. Smartphones with a built-in accelerometer and GPS-positioning function are popular in the general public and a good tool to collect daily steps without affecting people's lifestyles, which has great prospects in personal health management. WeChat-counted steps combined with self-conditioning can enhance physical activity management.

## Strengths and limitations

This study has several strengths. One of the strengths is that all data were collected under free-living conditions. Participants maintained their daily routines, which is difficult to replicate in controlled environments. Another strength is that the same participants were measured at multiple time points. A third strength is that it analyzed the influence of different conditions and demographic characteristics on consistency. Furthermore, health-related outcomes were measured, and the verification of WeRun step counts was examined more comprehensively, which makes the results more reliable.

One limitation of this study is that the sample size was not large. Second, there was no record of the time for which the participants wore the ActiGraph-GT3X accelerometer or carried smartphones in a day. Third, the use of the ActiGraph-GT3X accelerometer as the gold standard (43) could be seen as a limitation, but it is the reference tool for assessing physical activity in real life for 21 days and has well-established validity (44). Fourth, the long and unequal interval between the second and third measurement may affect the results of the study. Finally, according to the research results of Mitesh et al., there is a difference in the step measurement between the Android and iOS operating systems of smartphones (45), and there is a lack of investigations on step count accuracies measured by different smartphone brands, models, and operating systems.

## Conclusion

Under free-living conditions, the steps tracked by the WeChat smartphone app are highly correlated with the number of steps monitored by the ActiGraph-GT3X accelerometer, which is a reliable tool for measuring steps in daily life. However, different demographic characteristics (e.g., age, gender, education) and conditions can influence the accuracy of the WeChat app. The steps measured by the WeChat app may not replace the role of the moderate-to-vigorous intensity physical activity measured by the ActiGraph-GT3X accelerometer in predicting body composition.

## Data availability statement

The datasets presented in this article are available from the corresponding author on reasonable request.

## Ethics statement

The studies involving human participants were reviewed and approved by the Shanghai Municipal Center for Disease Control and Prevention Ethical Review Committee (ChiCTR900023813). The patients/participants provided their written informed consent to participate in this study.

## Author contributions

YJ, TL, JW, and YS conceptualized the idea. YJ, TL, and MC obtained funding. QYao and SS conducted data analysis. QYao drafted the paper. All authors participated in reviews, interpretation of analysis results, and critical revision of the paper. All authors approved the final version for submission.
